# Short Term Intrarectal Administration of Sodium Propionate Induces Antidepressant-Like Effects in Rats Exposed to Chronic Unpredictable Mild Stress

**DOI:** 10.3389/fpsyt.2018.00454

**Published:** 2018-09-27

**Authors:** Jianguo Li, Luwen Hou, Cui Wang, Xueyang Jia, Xuemei Qin, Changxin Wu

**Affiliations:** ^1^Laboratory for Microbiome Sciences, Institute of Biomedical Sciences, Shanxi University, Taiyuan, China; ^2^Key Laboratory of Chemical Biology and Molecular Engineering of Ministry of Education, Shanxi University, Taiyuan, China; ^3^Modern Research Center for Traditional Chinese Medicine, Shanxi University, Taiyuan, China

**Keywords:** propionate, antidepressant-like, metabolomics, Analysis of variance (ANOVA) Simultaneous Component Analysis, neurotransmitter

## Abstract

Depression has been correlated with metabolic disorders, and the gut microbiota and its metabolites have been reported to be key factors affecting metabolic disorders. Several metabolites generated by the gut microbiota have been reported to exert antidepressant-like effects, including the short chain fatty acid (SCFA) butyrate. However, recent work has suggested that the abundance of butyrate is not significantly changed in neither human nor experimental animals with depression, and butyrate has been reported to decrease upon the administration of prebiotics with antidepressant-like effects. Supplementation of endogenous metabolites that are unchanged in depression may induce additional metabolic disorders and may lead to poorer clinical outcomes. However, the endogenous metabolites that are imbalanced in depression may include several antidepressant candidates that could circumvent these problems. In this study, we used GC-MS spectrometry to study the fecal metabolome of rats under Chronic Unpredictable Mild Stress (CUMS). We carried out static and dynamic metabolomics analyses to identify the differential metabolites between the CUMS rats and control rats. We identified propionic acid, rather than butyric acid, as a differential metabolite of the CUMS rats. Consistent with this, a 1-week intrarectal administration of sodium propionate (NaP, the salt form of propionic acid) induced antidepressant-like effects and partially rebalanced the plasma metabolome. The antidepressant-like effects of NaP were correlated with differential rescue of neurotransmitters in the prefrontal cortex, which may be achieved through the reduction of catabolism of noradrenaline, tryptophan and dopamine, rather than serotonin. These findings support NaP as a potential candidate in fighting depression by administering an endogenous metabolite.

## Introduction

Depression is a widespread psychiatric disorder that is characterized by persistent depressive mood and anhedonia (1). Although the pathophysiology of depression is not yet completely understood, several hypotheses have been put forward, including lack of monoaminergic neurotransmitters, hyperactivity of the hypothalamic- pituitary-adrenal axis, chronic low-grade inflammation, and others (1, 2). Metabolic disorder was recently reported to be a novel feature of depression (3). Imbalanced metabolic states were observed in the serum (4), liver (5), and gut (6) of depressed patients and in experimental animals (7). In addition, restoring the metabolic state of these in experimental animal models has been associated with improvement of depression (8, 9), and several endogenous metabolites have been identified as potential biomarkers for depression (10–13).

The gut microbiota and its metabolites have also been reported to be important factors affecting depression (7, 14, 15). Fecal transplantation from depressed patients can transfer depressive symptoms to recipient animals (5, 16). Gut metabolites were correlated with a distinct liver metabolome in depressed mice (5), and it has been suggested that some metabolites have the potential to alleviate depression (17). One gut metabolite, butyrate, was reported to have antidepressant-like effects (18–20). However, there is little variation in the abundance of butyrate in the serum, liver, or gut of depressed individuals compared to healthy controls (21, 22). Moreover, supplementation with prebiotics that exhibit antidepressant-like effects decreased the abundance of butyrate in cecum of experimental animals (23), suggesting that butyrate may instead act to worsen depression symptoms. Therefore, caution should be taken when selecting an endogenous metabolite as an antidepressant candidate. To avoid inducing additional metabolome imbalances, candidate endogenous metabolites must therefore be evaluated for any potential influence on the depressed metabolic state before their antidepressant-like effects are investigated. Metabolites that typically show a reduced abundance in depression provide an alternative shortcut in the selection of endogenous metabolites as antidepressant candidates (21–24).

Metabolomics has been shown to be a powerful tool in searching for disease-specific metabolites (25, 26). LC-MS, GC-MS, and NMR are three of the most popular analytical platforms for metabolomics investigations (27, 28). Due to the sophisticated library of metabolite standards and the excellent coverage of non-polar small compounds, GC-MS spectrometry has been widely applied in metabolomics studies of drug pharmacology and disease pathophysiology (29, 30). Dozens of reports have used metabolomics to investigate the imbalanced metabolic state and the differential metabolites associated with depression (31–34). However, most of the current studies are limited to static metabolomics of cross-sectional data, which ignore the complicated dynamic development of depression (35).

Dynamic metabolomics was developed to overcome the above limitations of static metabolomics (36–38). Analysis of variance (ANOVA) Simultaneous Component Analysis (ASCA), one dynamic metabolomics strategy, accounts for time, phenotype, and the interactions between time and phenotype; divides metabolomics data into effect matrices; and reveals time-dependent trends through separate multivariate study of these effect matrices (39, 40). ASCA-based dynamic metabolomics has been successfully applied to evaluate the metabolic effects of energy-restricted intervention (41), to provide urinary metabolic profiling of a rat model of postnatal stress (42), and to evaluate the effect of amyloid peptide on hippocampal and serum metabolism (43).

This study uses GC-MS spectrometry and a combination of static and dynamic metabolomics data analysis to study the typical gut metabolites of rats with depression-like behaviors. Propionic acid was observed by both static and dynamic metabolomics data analysis, and intervention with NaP (the salt form of propionic acid) was carried out to study the effects of propionic acid on depression.

## Materials and methods

### Animals and reagents

Male Sprague-Dawley rats (weighing 200 ± 10 g) were purchased from Beijing Vital River Laboratories Co. (SCXK (Jing) 2011-2012). Rats were housed 10 per cage with free access to water and food, under controlled feeding conditions of temperature (25±1°C), humidity (45 ± 15%), and light (lights on at 8:00 a.m., 12-h day/night switch). Rats were allowed a 1-week adaption to the new environment before further experiments. The rats were randomly assigned into two groups: the Control group, and the CUMS group. The Control group contained 10 rats. The CUMS group contained 40 rats, and the success of CUMS modeling was evaluated by behavior tests at the fourth week. The rats without typical depressive-like behaviors were excluded (25% of the CUMS group). The successful modeled rats were then randomly separated into three groups: the CUMS group, the PBS group (CUMS+PBS administration), the NaP group (CUMS+NaP administration). The rats in each group were then filtered by the outliers of experimental results, and six rats per group was finally selected. The experimental results for each group were then backtracked. All experimental procedures were approved by the Committee on Animal Research and Ethics of Shanxi University.

Neurotransmitter standards: 5-hydroxytryptamine (5-HT), Kynurenine (KYN), 3-methoxytyramine (3-MT), gamma-aminobutyric acid (GABA), 3,4-dihydroxyphenylacetic acid (DOPAC), norepinephrine (NE), 5-hydroxyindole acetic acid (5-HIAA), Tryptophan (TRP), 3-hydroxykynurenine (3-HK), 3-hydroxyanthranilic acid (3-HAA), were purchased from Sigma-Aldrich (St. Louis, MO, USA). Dopamine (DA) was acquired from Dr. Ehrenstorfer GmbH (Augsburg, Germany). Homovanillic acid (HVA) and the derivatization reagent dansyl chloride were purchased from Tokyo Chemical Industry Co. Ltd. (Tokyo, Japan). Formic acid, acetone, methanol and acetonitrile (LC-MS grade) were obtained from Merck (Darmstadt, Germany). Standards for short chain fatty acids (SCFAs, acetic acid, propionic acid, butyric acid) were purchased from Sigma-Aldrich (St. Louis, MO, USA). All solvents were HPLC grade or above.

### Chronic unpredictable mild stress (CUMS) model

CUMS modeling was performed with a previously described protocol (44). Briefly, rats were individually housed and subjected to no more than 4 of the following stressors every day in a random order for 5 weeks: swimming in 4°C water for 5 min, foot-shock for 2 min, tail clamp for 2 min, subject to noise for 3 h, water deprivation for 24 h, food deprivation for 24 h, subject to a temperature of 45°C for 5 min. The above stressors were imposed separately, and each rat received no more than one stressor simultaneously. If a rat received a stressor of water deprivation or food deprivation, no more stressor was imposed to this rat at the same day. Every five rats of the control group was housed together, and received none of the above mentioned stressors. Fecal samples were collected with metabolic cage every week. After the experiment, rats were sacrificed after ethyl carbamate anesthesia. Blood samples were acquired through the arteria cruralis. EDTA anticoagulant-treated blood samples were centrifuged at 4°C, 3,000 rpm for 15 min, and the supernatants were split into three aliquots and stored at −80°C.

### NaP administration

**Figure 1:**
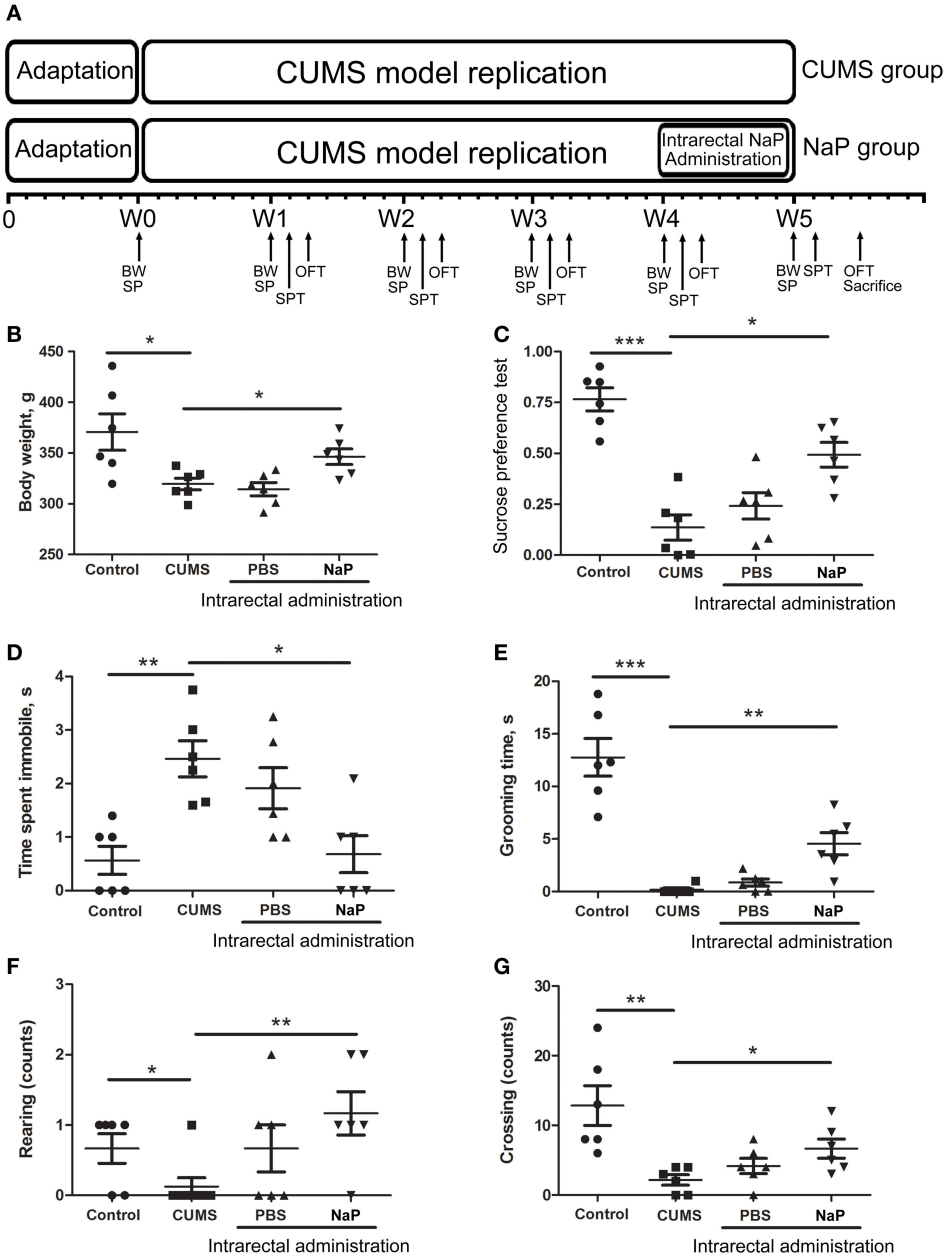
Experimental procedures **(A)** and results of behavioral tests **(B–G)** in this study. The rats were randomly assigned into two groups: the Control group, and the CUMS group. The Control group contained 10 rats. The CUMS group contained 40 rats, and the success of CUMS modeling was evaluated by behavior tests at the fourth week. The successful modeled rats were then randomly separated into three groups: the CUMS group, the PBS group (CUMS+PBS administration), the NaP group (CUMS+NaP administration). The rats in each group were then filtered by the outliers of experimental results, and six rats per group was finally selected. The experimental results for each group were then backtracked. CUMS modeling was performed over 5 weeks (W1–W4), and intrarectal administration of NaP was carried out during the last week (W5). Body weight **(B)**, sucrose preference rate **(C)**, and indices of OFT **(D–G)** were measured for CUMS and control animals every week during this period, and the measurements in the fifth week are shown. BW, body weight; SP, sample collection; SPT, sucrose preference test; OFT, open field test. One-way ANOVA was used to determine the statistical significance of differences between groups. ^*^p < 0.05, ^**^p < 0.01, and ^***^p < 0.001.

NaP was administrated intrarectally every day for 1 week from the beginning of the 5th week (Figure 1A). To reduce variation between individuals, each rat in the NaP group received 1 mL of NaP (200 mmol/L) in PBS (pH 7.4), while rats in the PBS group received an equal volume of PBS.

### Behavioral tests

#### Body weight measurement

The body weights of rats in both of the CUMS group and the control group were measured at 9 a.m. on days 0, 7, 14, 21, 28, 35, and 42 of the CUMS model.

#### Sucrose preference test (SPT)

Exposure to 1% sucrose solution for 24 h was carried out before SPT to avoid neophobia. To test for sucrose preference, each rat was provided with a bottle containing a 1% sucrose solution and a second bottle containing tap water for 4 h. Consumption of sucrose and water were recorded. The sucrose preference rate was calculated as: sucrose consumption (g)/ (sucrose consumption (g) + water consumption (g)).

#### Open-field test (OPT)

OPTs were performed once a week in a custom-made black metal cage (100 × 100 × 40 cm), with the bottom divided into 25 equal sectors by white stripes. To avoid influence of confounding factors (the environment and animals' emotional and physical state, etc.), OPT was performed in the morning of the same day of the weeks during the CUMS modeling. Each rat was gently placed into the central square and monitored for 5 min. The immobility time, grooming time, rearing counts, and crossing counts were recorded. The bottom of the open field was cleaned before each OPT.

#### GC-MS spectrometry

GC-MS spectrometry was conducted as previously described (44), with some modifications. Briefly, 200 mg of dried feces was homogenized in 500 μL water, and 400 μL acetonitrile was added into the centrifuged supernatant to precipitate protein. The second supernatants were then thoroughly dried under nitrogen and re-suspended in 30 μL Pyridine-methoxy amino acid salt solution (15 mg/mL). The solution was subsequently incubated at 70°C for 1 h, 50 μL of N,O-bis(trimethylsilyl)tri- fluoroacetamide (including 1% trimethylchlorosilane) was added to the solution, and the samples were incubated at 40°C for 1.5 h. One microliter of each analyte was injected in a (10:1) split mode into a trace gas chromatograph coupled with a Polyris Q Ion Trap mass spectrometer (Thermo Fisher Scientific, MA, USA). Separation of the ECF derivatives was conducted with a DB-5MS capillary column (30 m × 250 μm i.d., 0.25 μm film thickness, Agilent J & W Scientific, CA, USA). Helium was employed as carrier gas at a constant flow rate of 1.0 mL/min. The oven temperature was first held at 80°C for 3 min, ramped to 140°C at a speed of 7°C/min, held at 140°C for 4 min, ramped to 180°C at a speed of 4°C/min, held at 180°C for 6 min, then ramped to 280°C at a speed of 5°C/min, held at 280°C for 2 min. The mass data were collected in a full scan mode from m/z 50 to 650. Compounds were identified by comparison of mass spectra with the standards in National Institute of Standards and Technology (NIST) library (version 2.0). The Human Metabolome Database (HMDB) (http://www.hmdb.ca) was employed for further reference. The identified metabolites were validated with commercially available analytical standards. The raw GC-MS result files were converted into Net-CDF format and processed using XCMS with default settings.

### Quantitation of neurotransmitters and SCFAs

Simultaneous UHPLC-ESI-MS/MS quantitation of 12 neurotransmitters in the prefrontal cortex (PFC) of rats was carried out as previously described (45) with some modifications. Briefly, 30–50 mg of the PFC was homogenized and precipitated with methanol, the supernatants were dried under nitrogen and reconstituted with the initial mobile phase. UHPLC-ESI-MS/MS was performed on a Thermo Scientific Dionex Ultimate 3000 RSLC system combined with a Thermo Q Exactive Orbitrap mass spectrometer. The analytes were separated with a Thermo Hypersill GOLD (2.1 × 100 mm, 1.7 μm) column. The mobile phase consisting of phases A [water: formic acid (99.9: 0.1, v/v)] and B [acetonitrile: formic acid (99.9: 0.1, v/v)] was applied with a gradient elution at a flow rate of 0.3 mL/min: linear increase from 0 to 20% B in 3 min; hold at 60% B for 3 min; linear increase from 60 to 80% B in 4 min; linear increase from 80 to 95% B in 3 min; hold at 95% B for 4 min. ESI-MS/MS conditions were set as follows: gas temperature 350°C, sheath gas flow rate 46, capillary voltage 3000 V, nebulizer pressure 35 ps. MS acquisitions were performed in PRM (Parallel Reaction Monitoring, also known as Targeted-MS/MS) mode. The calibration curves for each analyte were obtained by linear regression analysis with 1/x2 weighting factor, which contained 10 data points covering a linear range of 0.02–20 ng. Data acquisition and analysis were performed with Thermo Xcalibur 2.2 software. Simultaneous UHPLC-ESI-MS/MS quantitation of SCFAs were performed as previously described (46).

### Data analysis

#### Static metabolomics

The GC-MS spectrometry generated data was introduced to SIMCA-P 13.0 (Umetrics AB, Umea, Sweden) for static multivariate analysis. Principal component analysis (PCA) was used to explore the natural separation of metabolomes between the study groups. Orthogonal Projection to latent structure-discriminate analysis (OPLS-DA) was used to investigate the difference between groups by incorporating known classification information. The results were presented with S-Plot, in which each plot represented one metabolite ion. The distance of a plot from the origin represented the contribution of the corresponding metabolite to the separation. Metabolites with Variable Importance in Projection (VIP) values greater than 1 in the established OPLS-DA model and *P* < 0.05 in an independent-samples *t*-test were considered to be differential metabolites contributing to the separation of the study groups.

#### Dynamic metabolomics

The metabolomic datasets from all five time points of the CUMS model (Figure 1A) were combined, peak aligned and then introduced to the MetaboAnalyst web portal (40). The combined dataset was normalized to constant sum, transformed with log transformation and Pareto scaling before further analysis. ASCA was applied to split the original dataset into subsets describing the variations of phenotypes, the variations of time, and their interactions. SPE (Squared Prediction Error) and Leverage were proposed to evaluate the fitness of the ASCA model. SPE (derived from residuals) was used to test the fitness of a model for the metabolite. Leverage (derived from loadings) was used to evaluate the importance of a metabolite to the model. Variables with high Leverage values and low SPE values were considered to have significant contributions to the model.

Group data were expressed as mean ±S.E.M. Statistical analyses were performed with independent *t*-test in SPSS 22.0 (Chicago, USA), and values of *P* < 0.05 were considered statistically significant.

## Results

### Data acquisition quality and model validations for the metabolomics data analyses

Quality controls (QCs) were used to evaluate the performance of the analytical system and to monitor the robustness of sample preparation and the stability of instrument analysis. QCs for the GC-MS spectrometry were prepared by pooling equal aliquots of fecal samples. The first five QCs were tested before the analysis to stabilize the analytical system, and the acquired results were removed prior to data processing. Tight clustering of the QCs in PCA scores was observed (Supplementary Figure 1), suggesting a good reproducibility of the metabolomics experiments.

Model validations for static and dynamic metabolomics were also performed. For the static metabolomic analysis, a validation plot for the arrangement analysis of the selected PLS-DA model was generated (Supplementary Figure 2). The resulting correlation (*R*^2^ = 0.553) demonstrated that the selected model could explain 55.3% of the total variables in the static metabolomics data, while the observed *Q*^2^ = 0.838 suggested that the model had good predictive power. A permutation-based significance test was performed to validate the model selected for the ASCA dynamic metabolomics analysis. Significance levels of *P* < 0.05 for the phenotype, the time, and the interaction between phenotype and time were observed (Supplementary Figure 3B), demonstrating an acceptable fitness of the selected model for ASCA analysis.

### CUMS model induced depression-like phenotypes

CUMS is a widely accepted method for modeling depression in experimental animals (8, 18, 47–49), and there is growing evidence for correlations between depression and the serum or urine metabolome (31, 50). To further investigate the correlation between the gut metabolome and depression, we replicated the CUMS rat model and monitored changes in behavioral indices and neurotransmitters in the PFC (Figure 1A). Compared to the healthy controls, CUMS rats suffered a significant decrease in body weight (Figure 1B) and sucrose preference rate (Figure 1C) in the fifth week. In parallel, OFTs showed significant variations in behavioral indices including immobility time, grooming time, rearing counts, and crossing counts in the fifth week (Figure 1D–G). We also observed significant decreases in several neurotransmitters (including 5-HT, 5-HIAA, NE, DA, TRP, 3-HAA, 3-HK) in the PFC of CUMS rats (Figure **4**). These results suggested that the CUMS model was successfully replicated.

### Metabolomic trajectory analysis revealed distinct fecal metabolomes in CUMS rats

**Figure 2:**
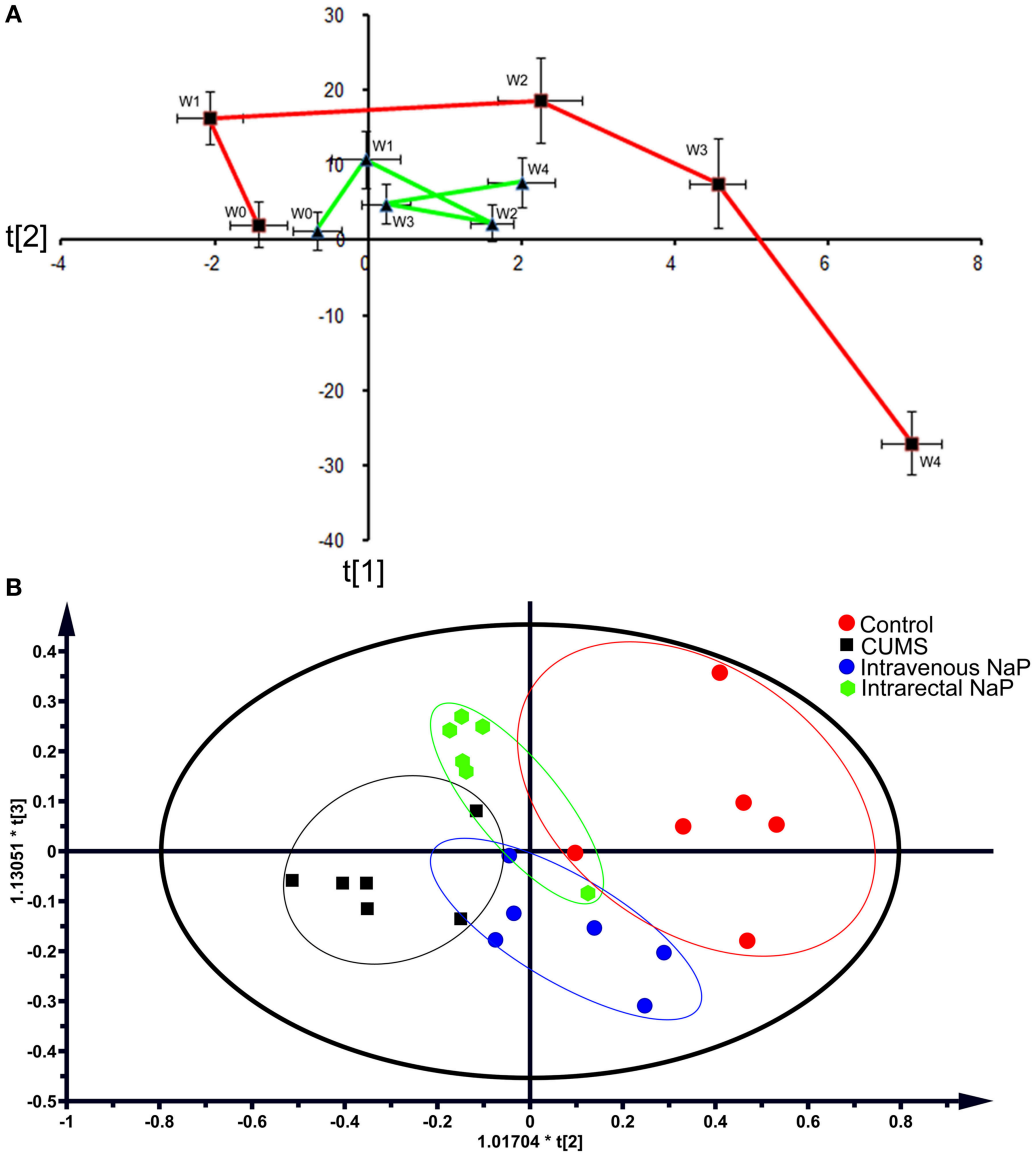
PCAs of the fecal metabolomes for the time-coursed trajectories **(A)** and the effects of NaP administration **(B)**. For the time-coursed metabolomics trajectories analysis, dots represent the average metabolic status, and bar lines indicate the standard deviations of PC1 (horizontal axis) and PC2 (longitudinal axis) in the PCA model. The red and green lines represent the CUMS and the control groups, respectively. For the PCA plots of the effects of NaP administration, the red dots represent the control animals, the black dots represent the CUMS animals, the green dots represent the CUMS animals that received intrarectal administration of NaP, and the blue dots represent the CUMS animals that received intravenous administration of NaP with the same dose of the intrarectal administration group.

Metabolomic trajectory may represent the dynamics of host responses to environmental changes. To investigate the dynamic changes in the gut metabolome during the development of depression, fecal metabolomic datasets generated from the first five time points (W0–W4) of the CUMS model (Figure 1A) were analyzed with PCA. The mean scores of the first two principal components (PCs) of PCA were used to infer the time-course trajectories of fecal metabolome. We observed dramatically different paths of CMUS animals and healthy controls (Figure 2A). The CUMS rats exhibited a large response, while those of the control groups clustered tightly during the time course. This distinct metabolomic trajectory of the CUMS rats indicates a possible correlation between the gut metabolome and depression. A further investigation of this relationship will be significant in providing a clearer understanding of the pathophysiology of depression. Because the three-way high-dimensional dataset (multi-variables, multi-subjects and multi-time points)), time-resolved metabolomics is too complex to investigate using any single method. To reach a more objective conclusion, we therefore carried out static and dynamic metabolomics data analysis from multiple perspectives in subsequent experiments.

### Static metabolomics analysis observed propionic acid as a differential metabolite of the CUMS rats

**Figure 3:**
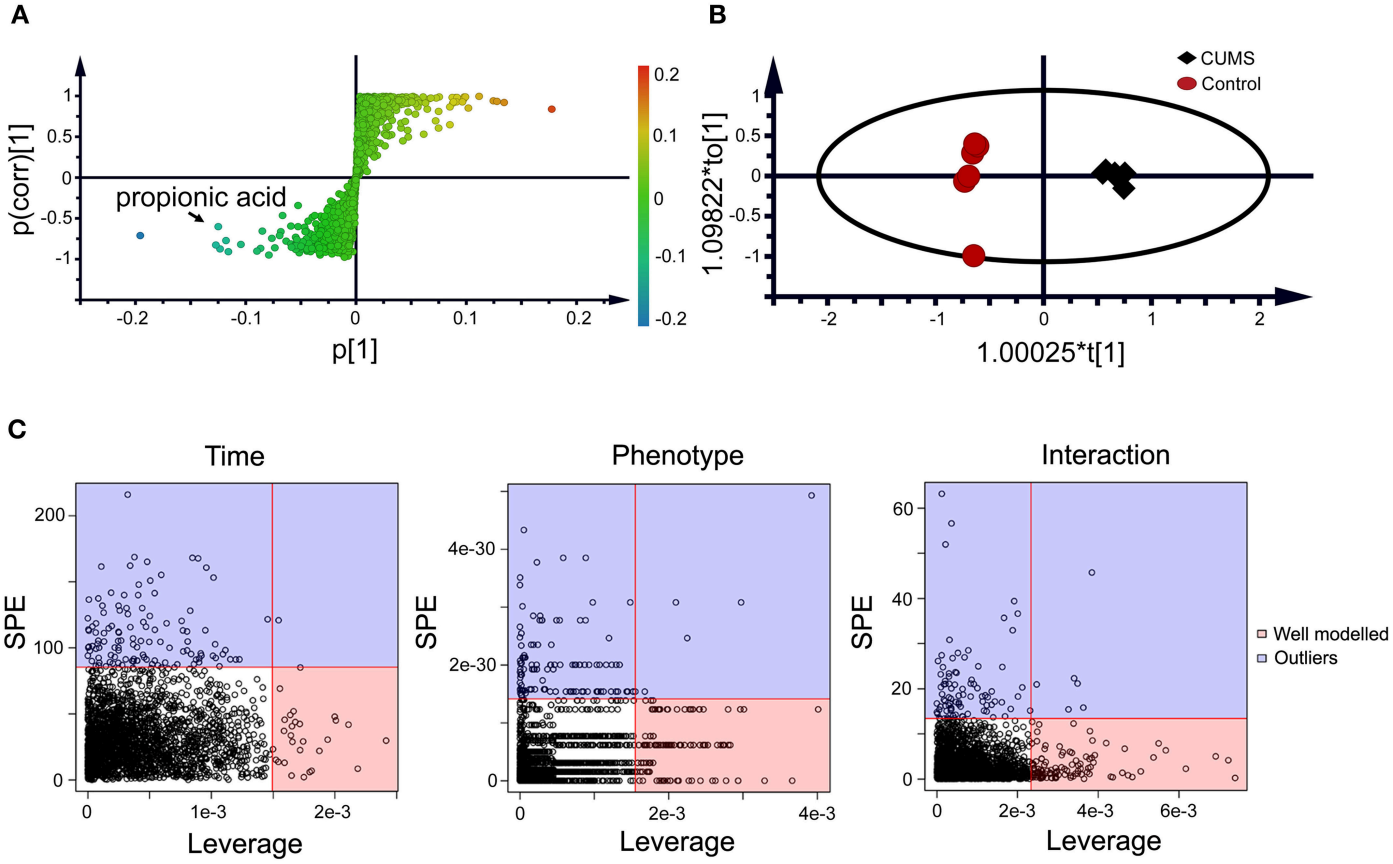
Static **(A,B)** and dynamic **(C)** metabolomics analyses of the fecal metabolomes collected in the 5th week of CUMS modeling. S-Plot **(A)** and OPLS-DA plot **(B)** were used to visualize the static metabolomics analysis. Metabolites with VIP>1 and *p* < 0.05 were considered to be differential metabolites. Only propionic acid was labeled in the S-plot; please refer to Table 1 for detailed information on all differential metabolites identified via the static metabolomics analysis. ASCA **(C)** was used to analyze in the dynamic metabolomics data. Metabolites with a high leverage value and a low SPE value were considered to be differential metabolites (the well-modeled group); please refer to Table 2 for detailed information on all of the differential metabolites identified via ASCA dynamic metabolomics analysis.

Within the 2,511 spectral features generated from the fecal metabolome of all five time points of CUMS animals, 45 metabolites were putatively validated (Supplementary Table 1). Static metabolomics analysis was carried out based on the dataset generated from the rats with successful replicated CUMS model (at the fifth week of treatment) and the corresponding healthy controls. S-Plot (Figure 3A) and OPLS-DA (Figure 3B) analyses were applied to identify differential metabolites differentiating the CUMS rats and the healthy controls. A total of 15 differential metabolites were identified (Table 1), including 2 fatty acids, 2 diatomic fatty acids, 10 amino acids, and glycerol. Interestingly, propionic acid was the only SCFA differentiating the gut metabolome of the CUMS rats from that of the healthy controls, while no changes were observed in acetic acid or butyric acid. These findings suggested that propionic acid is a differential metabolite in the fecal metabolome of the depression-like animal model.

**Table 1:** Typical fecal metabolites of the CUMS rats.

**Metabolite**	**VIP**	***P***
Lactic acid	1.22	1.00E-02
Succinic acid	1.42	3.35E-04
Glutaric acid	1.72	1.00E-04
Methionine	2.45	1.87E-05
Threonine	3.10	2.99E-05
Proline	3.23	7.10E-04
Leucine	3.45	3.89E-04
Valine	3.89	1.56E-03
Glycine	4.65	6.22E-04
Serine	5.02	1.23E-05
Isoleucine	5.05	1.94E-05
Alanine	5.87	1.22E-04
Glycerol	6.24	4.65E-04
Aspartic acid	6.33	8.73E-04
Propionic acid	6.55	2.32E-05

*VIP, variable importance in projection*.

### ASCA dynamic metabolomics analysis confirms propionic acid as a differential metabolite of CUMS rats

To further validate the findings of static metabolomics analysis, we carried out ASCA dynamic metabolomics analysis. ASCA analysis split the multilevel, time-coursed metabolomic datasets into subsets describing the variations between rats of the CUMS group and the control group, the variations along time-scale, and their interactions. The metabolomic datasets were normalized before further investigations (Supplementary Figure 3A). We observed an excellent fitness of the model selected for the ASCA analysis, with high significance levels (*p* < 0.05) of phenotype, time and their interactions generating from a permutation test (Supplementary Figure 3B). Leverage/SPE was used to identify significant spectrometric features associated with a specific factor. Features with high Leverage values and low SPE values were included in the “well-modeled” group in the ASCA analysis. In all, 15 metabolites were identified from the greatly altered (and well-modeled) features over time, phenotype or their interactions (Figure 3C, see details in Table 2 and Supplementary Table 2). This analysis, like the static metabolomics analysis, identified propionic acid as a significant metabolite discriminating the CUMS rats and the healthy controls. To validate the findings from metabolomics analyses, we then determined the abundance of SCFAs in plasma. Propionic acid was the only SCFA with significantly changed abundance in the plasma of the CUMS rats (Figure **5**). The collective findings from both the static and the dynamic metabolomics analyses led us to hypothesize that propionic acid may play a role in the pathophysiology of depression.

**Table 2:** Typical metabolites identified by the ASCA dynamic metabolomics analysis.

**Model**	**Metabolite**	**Leverage**	**SPE**
Time	Lactic acid	2.00E-03	4.79E+00
	Valeric acid	2.11E-03	4.18E+00
	Glycerol	1.55E-03	6.91E+00
	Isoleucine	1.59E-03	3.71E+00
	Serine	1.75E-03	2.08E+00
	Valine	1.65E-03	4.06E+00
Phenotype	Lactic acid	2.30E-03	3.65E-30
	Acetic acid	2.41E-03	1.23E-30
	Hexanoic acid	1.96E-03	6.16E-31
	Phosphoric acid	2.49E-03	6.16E-31
	Succinic acid	2.15E-03	6.16E-31
	Valeric acid	2.22E-03	1.23E-30
	Malic acid	2.04E-03	6.16E-31
	Glycerol	2.29E-03	6.16E-31
	Glycine	1.93E-03	6.16E-31
	Valine	1.79E-03	7.70E-31
	Sarcosine	2.94E-03	6.16E-31
	Serine	1.74E-03	1.54E-31
	Threonine	1.74E-03	3.08E-31
	Alanine	2.83E-03	6.16E-31
	Aspartic acid	1.62E-03	1.54E-31
	Pyrimidine	1.86E-03	1.23E-30
Interaction	Lactic acid	3.02E-03	9.26E-01
	Propanoic acid	2.60E-03	1.77E+00
	Valeric acid	3.13E-03	5.92E+00
	Malic acid	3.04E-03	7.22E+00
	Glycerol	3.35E-03	5.81E+00
	Leucine	2.38E-03	3.79E-01
	Glycine	2.48E-03	1.44E+00
	Pyrimidine	2.83E-03	2.01E+00
	Serine	2.60E-03	2.22E-01
	Threonine	2.52E-03	1.23E+00
	Methionine	6.91E-03	5.02E+00
	Valine	2.48E-03	8.39E+00
	Sarcosine	3.23E-03	1.87E+00

### Short-term intrarectal administration of NaP induces antidepressant-like effects

Because we had identified and validated propionic acid as a typical fecal metabolite of the CUMS rats using static and dynamic metabolomics analysis and because the abundance of propionic acid was decreased in the plasma of CUMS rats, we next investigated the role of NaP (the salt form of propionic acid) in the pathophysiology of depression. To do this, we introduced NaP to the CUMS rats via intrarectal administration. A 1-week intrarectal administration of NaP rescued the loss of body weight and sucrose preference phenotypes of the CUMS rats (Figures 1B,C). The administration of NaP also rescued the behavioral indices in the OFT (Figures 1D–G) and restored plasma levels of propionic acid (Figure **5**). In addition, NaP administration partially rescued the imbalanced plasma metabolomes of CUMS rats (Figure 2B). These results suggested that intrarectal administration of NaP has short-term antidepressant-like effects.

**Figure 4:**
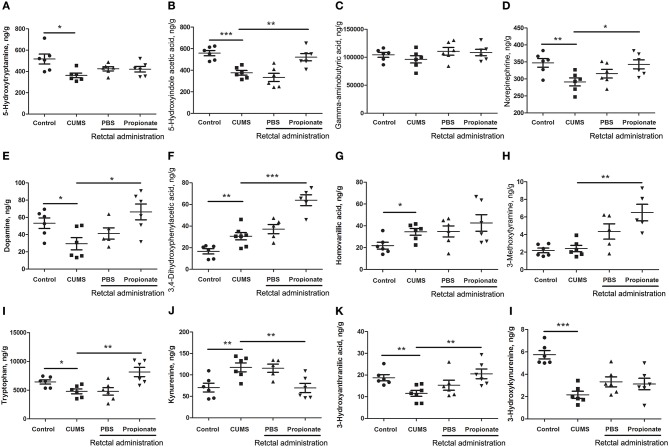
The abundance of neurotransmitters in the prefrontal cortex of rats exposed to CUMS and NaP. UHPLC-ESI-MS/MS was used to quantify neurotransmitters in the plasma collected in the 5th week of the CUMS animals. 5-HT **(A)**, 5-HIAA **(B)**, GABA **(C)**, NE **(D)**, DA **(E)**, DOPAC **(F)**, HVA **(G)**, 3-MT **(H)**, TRP **(I)**, KYN **(J)**, 3-HAA **(K)**, 3-HK **(L)** were quantified. One-way ANOVA was used to determine the statistical significance of differences between groups. ^*^p < 0.05, ^**^p < 0.01, and ^***^p < 0.001.

Neurotransmitter imbalance in the central nervous system has been associated with depression (51, 52). To further investigate the effects of NaP administration, we next determined the abundance of several neurotransmitters in the PFC of the CUMS rats (Figure 4). The decreased levels of NE, DA, TRP, 5-HIAA, and 3-HAA in the PFC of CUMS rats were restored by short term intrarectal NaP administration, but decreased 5-HT and 3-HK were not. Among the metabolites of DA, the increased abundance of DOPAC by CUMS was further up-regulated by NaP administration, but the abundance of HVA was not significantly changed, and the unchanged levels of 3-MT were significantly up-regulated. Because neurotransmitter metabolism has been correlated with the pathophysiology of depression (53) and with the antidepressant-like effects of endogenous metabolites (24), we then examined the effects of NaP administration on the turnover of the depression associated neurotransmitters (Table 3). The increased turnover of TRP to KYN (calculated as KYN/TRP) by CUMS was significantly down-regulated by NaP administration, while the increased turnover of DA to HVA (calculated as HVA/DA) was also restored by NaP administration; the unchanged turnover of 5-HT to 5-HIAA (calculated as 5-HIAA/5-HT) was not significantly influenced by NaP administration. In addition, the decreased turnover of KYN to 3-HK was significantly up-regulated by NaP administration. These results suggest that short-term intrarectal administration of NaP selectively restores the metabolism of neurotransmitters in the PFC of CUMS rats.

**Table 3:** Turnover of neurotransmitters in the PFC of rats under CUMS and intrarectal NaP administration.

	**Control**	**CUMS**	**Intrarectal administration**	
			**PBS**	**NaP**
5-HIAA/5-HT	1.11	1.04	0.98	1.28
KYN/TRP	0.011	0.0245^***^	2.41E-02	0.00878^###^
3-HAA/KYN	0.0029	2.60E-03	2.30E-03	2.50E-03
3-HK/KYN	0.092	0.02^**^	2.87E-02	0.0465^#^
HVA/DA	0.43	1.59^*^	1.44	0.49^#^
DOPAC/DA	0.31	1.51^**^	1.46	0.98
3-MT/DA	0.043	0.095^***^	1.03E-01	1.05E-01

^*^Significance value from Student-t-test between the Control group and the CUMS group.

* P < 0.05,

** P < 0.01,

*** P < 0.001.

#Student-t-test between the CUMS group and NaP administration group.

# P < 0.05,

### *P < 0.001*.

## Discussion

In the present study, propionic acid was identified by both static and dynamic metabolomics analyses as a differential metabolite in the fecal metabolome of CUMS rats. A 1-week intrarectal administration of NaP (the salt form of propionic acid) induced antidepressant-like effects, which reversed the depression-like behavior and selectively restored neurotransmitter levels in the PFC. These findings provide support for NaP as a promising potential alternative in fighting against depression using an endogenous metabolite.

Several endogenous metabolites have been reported to have antidepressant-like effects with distinctive action mechanisms (24, 54–57). The anti-depressant-like effects of lactate was reported to be associated with serotonin receptor trafficking, astrocyte function, neurogenesis, nitric oxide synthesis, and cAMP signaling (24). Agmatine has been reported to attenuate depression-like symptoms by modulating the nitrergic signaling pathway (54, 58). Oleoylethanolamide may exert its antidepressant-like effects through the regulation of brain-derived neurotrophic factor (BDNF) levels in the hippocampus and PFC, via antioxidant defenses, and by normalizing the hyperactivity of the hypothalamic-pituitary-adrenal (HPA) axis (55). One SCFA, butyrate, was also reported to have antidepressant-like effects with a multi-faced mechanism, including up-regulating the concentration of 5-HT in the brain, increasing the expression of BDNF and restoring blood-brain barrier impairments (18), acting as histone deacetylase inhibitor (HDACi) to influence microglial activation (56) or gene expression in the hippocampus (59), or acting as an N-Methyl-D-aspartate receptor (NMDA) enhancer (19).

**Figure 5:**
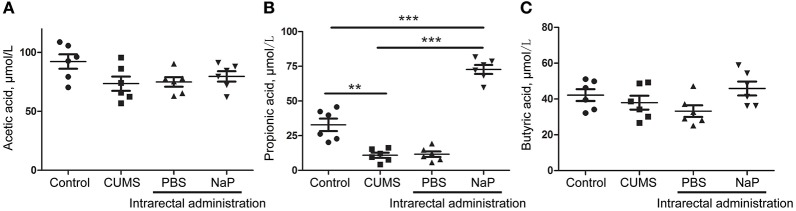
Quantitation of SCFAs in the plasma of rats at the 5th week of CUMS modeling. UHPLC-ESI-MS/MS was used for simultaneous quantitation of acetic acid **(A)**, propionic acid **(B)**, and butyric acid **(C)** from plasma of the control group, the CUMS group, the intrarectal PBS group, and the intrarectal NaP group. One-way ANOVA was used to determine the statistical significance of differences between groups. ^**^p < 0.01, and ^***^p < 0.001.

Although butyrate administration induced antidepressant-like effects, the abundance of butyrate (or butyric acid) was not significantly changed in depressed animal models (5) or humans (58) in previous reports or in the present study (Figure 5). Supplementation of these unchanged endogenous metabolites may lead to poorer outcomes for depression by inducing additional metabolome imbalance, due to the close associations between depression and metabolic disorders in serum (4), liver (5), feces (7), and the central nervous system (60). Nevertheless, another SCFA, propionic acid, was greatly altered in the feces and the plasma of our animal model with depression-like behaviors, which is consistent with a previous report (61). It was also reported that prebiotics with antidepressant-like effects may raise the abundance of propionate and reduce the abundance of butyrate in the cecum of animals with depression-like behaviors (23). Taken together, this study and previous work suggest that propionate may be a better candidate endogenous metabolite for antidepressant than butyrate.

The PFC is a key brain area that is responsible for working memory and emotional regulation (62). The antidepressant-like effects of NaP in this study are correlated with the levels of several neurotransmitters in the PFC. Neurotransmitter imbalance in the PFC is one of the classical pathophysiologies of depression (52, 63) and is associated with the effects of antidepressants (64). Accordingly, we found that NaP exerts antidepressant-like effects through selective rescue of neurotransmitter levels in the PFC. Intrarectal administration of NaP completely restored the levels of depression-associated neurotransmitters in the PFC, except 5-HT (Figure 4A). This is quite different from the observed effect of butyrate, which up-regulated the levels of 5-HT (18). Although the decreased 5-HT was not affected in this study, another neurotransmitter, NE, was completely rescued by short-term NaP administration (Figure 4D). It has been reported that NE is responsible for the quick onset effects of antidepressants (65), while 5-HT is correlated with the late-onset anti-depression effects of the neurotransmitter reuptake inhibitors (66). It is thus reasonable to hypothesize that the short-term, NaP-induced, quick-onset, antidepressant-like effects were correlated with the recovery of NE in the PFC of the CUMS rats.

The altered function of TRP metabolic pathway has been proposed as one of the crucial links between neurotransmitter dysregulation and the aberrant immune function associated with depression (67). Consistent with a previous report (68), we observed increased TRP turnover in the CUMS rats in this study, which increased the accumulation of KYN and further increased the production of several neurotoxic metabolites such as 3-HK (Figure 4I). In addition, NaP administration reduced the turnover of TRP by decreasing the production of KYN and its neurotoxic metabolites, which is consistent with the reported antidepressant effects of glycyrrhizic acid (69) and ketamine (70).

DA is another important neurotransmitter that is partially responsible for the anhedonia of depression. The decreased DA in the CUMS rats was rescued by NaP administration in this study, and the increased total catabolism of DA, as indicated by the ratio of HVA/DA (71) was decreased by NaP administration (Table 3). This is consistent with the reported antidepressant effects of paroxetine (72) and fluvoxamine (73). It is difficult to elucidate the major DA catabolism pathway that is affected by NaP administration, because the ratios of DOPAC/DA and 3-MT/DA were not significantly changed. NaP administration may decrease the up-regulated DA catabolism by exerting week effects on both the MAO (monoamine oxidase)-dependent oxidative pathway (DOPAC/DA) and the COMT (catechol-O-methyltransferase)-dependent methylation pathway (3-MT/DA) (74). These results may therefore suggest that NaP exerts its antidepressant-like effects partially by decreasing the turnover of DA.

As a limitation of this study, OPT is insufficient to some extent to represent the depressive-like behaviors. However, the CUMS modeling in this study was also supported by sucrose preference rate and neurotransmitters quantitation, representing key features of depression of anhedonia and neurotransmitters depletion, respectively. More behavior tests are needed to obtain more solid results in future similar studies. Another limitation of this study is lack of behavior data of the rats after NaP administration, which is valuable to evaluate the duration of the antidepressant-like effects. Future similar studies should pay attention on this point.

In conclusion, the present study demonstrated that propionic acid is a differential metabolite in CUMS rats via static and dynamic metabolomics analyses. Furthermore, we found that short term intrarectal administration of NaP induced antidepressant-like effects, possibly by up-regulating the abundance of NE and down-regulating the turnover of TRP and DA in the PFC of the CUMS rats. Future clinical investigations will be required to validate NaP as a novel endogenous metabolite antidepressant candidate.

## Ethics statement

This study was carried out in accordance with the recommendations of UFRN protocol No. 034/2014. The protocol was approved by the Committee on Animal Research and Ethics of Shanxi University.

## Author contributions

JL and XQ conception and design. JL, CWa, CWu and LH analysis and interpretation. JL, XJ, and XQ drafting the manuscript for important intellectual content.

### Conflict of interest statement

The authors declare that the research was conducted in the absence of any commercial or financial relationships that could be construed as a potential conflict of interest.

## Supplementary material

Supplementary Figure 1 PCA scores plot of the QCs and fecal samples within the run analyzed in GC-MS for evaluation of the data acquisition quality. QCs were prepared through pooling equal aliquots of fecal samples. The first five QCs were tested before the analysis to stabilize the analytical system, and the acquired results were removed prior to data processing. PCA analysis was carried out with SIMCA-P 13.0 (Umetrics AB, Umea, Sweden) after total area normalization. Each dot represents one sample (green) or one QC (red).

Supplementary Figure 2 Model validation plot of the static metabolomics data. The validation analysis was performed through pair-wise comparison of the fecal metabolomes from the CUMS group and the healthy control group at the 5th week of CUMS modeling. A 200 permutation was applied for the validation analysis.

Supplementary Figure 3 Data normalization **(A)** and model validation **(B)** of the dynamic metabolomics data. The effect of data normalization was shown **(A)**. Model validations were performed through permutations, as demonstrated by significance levels of *p* < 0.05 for the phenotype, time, and the interaction between phenotype and time.

Supplementary Table 1 Metabolites identified from fecal metabolome of rats in this study.

Supplementary Table 2 Typical metabolites identified by the ASCA dynamic metabolomics analysis.
