# Supplementary figures and images for: Short Term Intrarectal Administration of Sodium Propionate Induces Antidepressant-Like Effects in Rats Exposed to Chronic Unpredictable Mild Stress
Source: Front Psychiatry. 2018 Sep 27;9:454. doi: 10.3389/fpsyt.2018.00454 (PMC6170646; doi:10.3389/fpsyt.2018.00454)

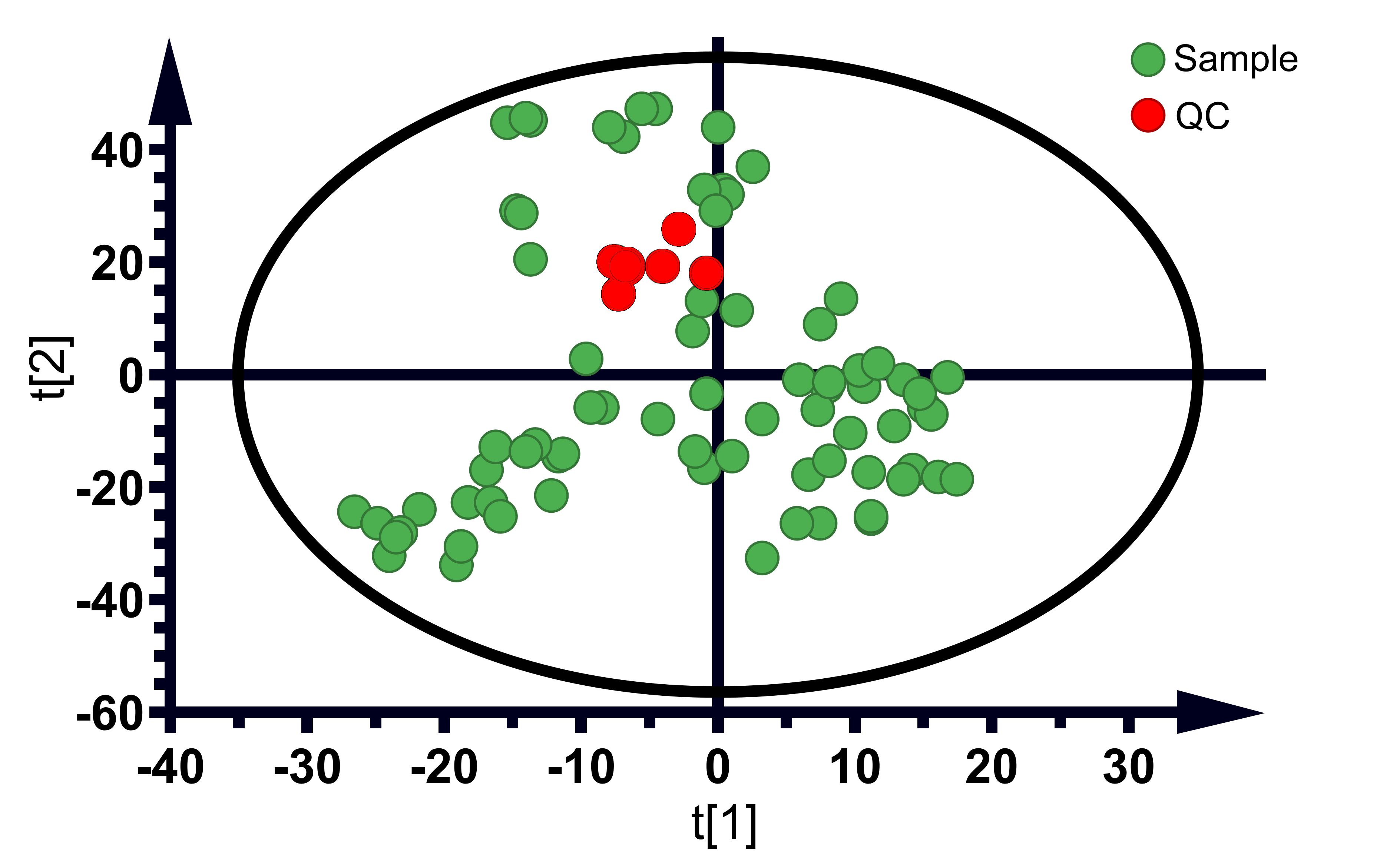

Supplement: Supplementary Figure 1 — PCA scores plot of the QCs and fecal samples within the run analyzed in GC-MS for evaluation of the data acquisition quality. QCs were prepared through pooling equal aliquots of fecal samples. The first five QCs were tested before the analysis to stabilize the analytical system, and the acquired results were removed prior to data processing. PCA analysis was carried out with SIMCA-P 13.0 (Umetrics AB, Umea, Sweden) after total area normalization. Each dot represents one sample (green) or one QC (red). [file Image_1.TIF]

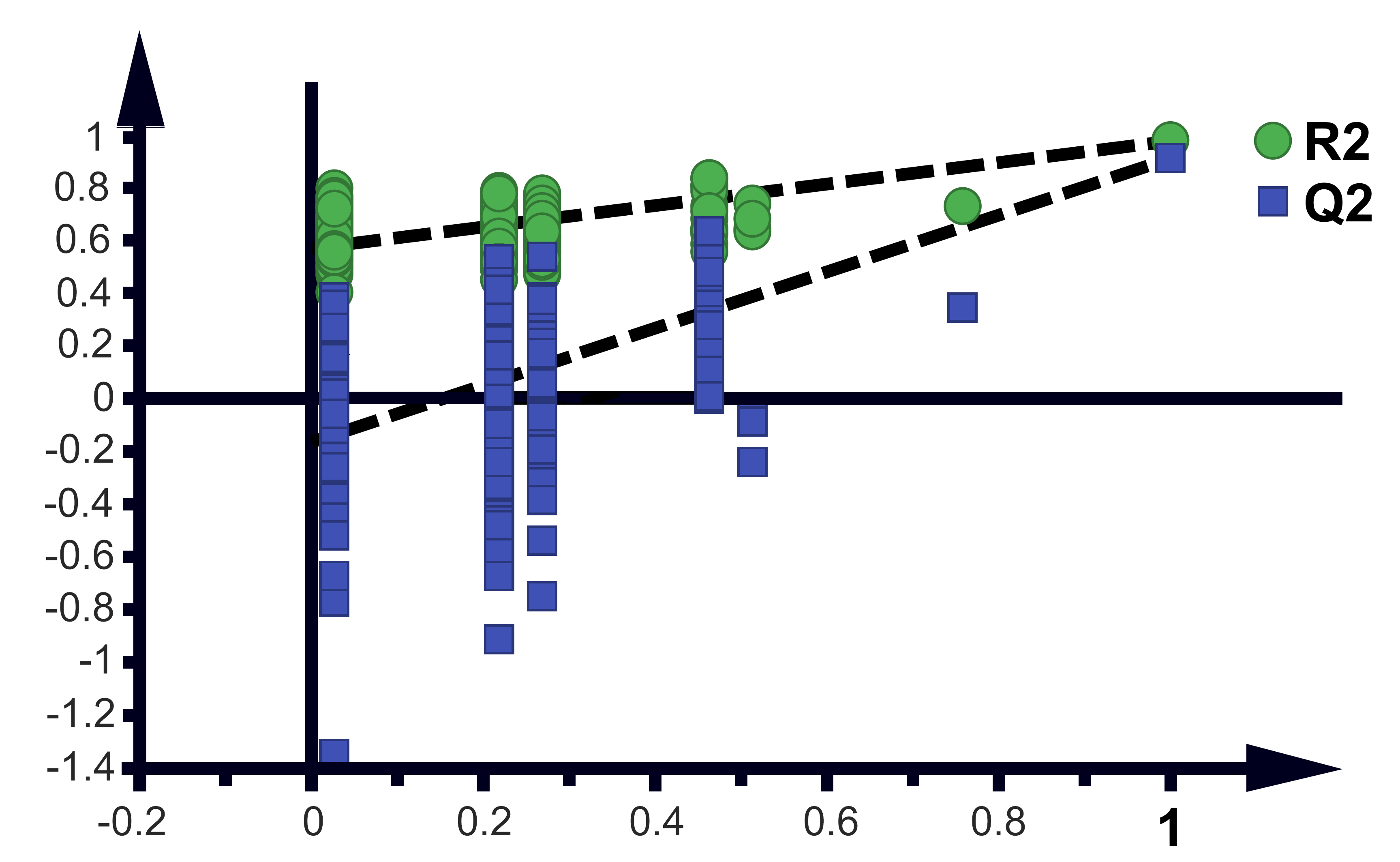

Supplement: Supplementary Figure 2 — Model validation plot of the static metabolomics data. The validation analysis was performed through pair-wise comparison of the fecal metabolomes from the CUMS group and the healthy control group at the 5th week of CUMS modeling. A 200 permutation was applied for the validation analysis. [file Image_2.TIF]

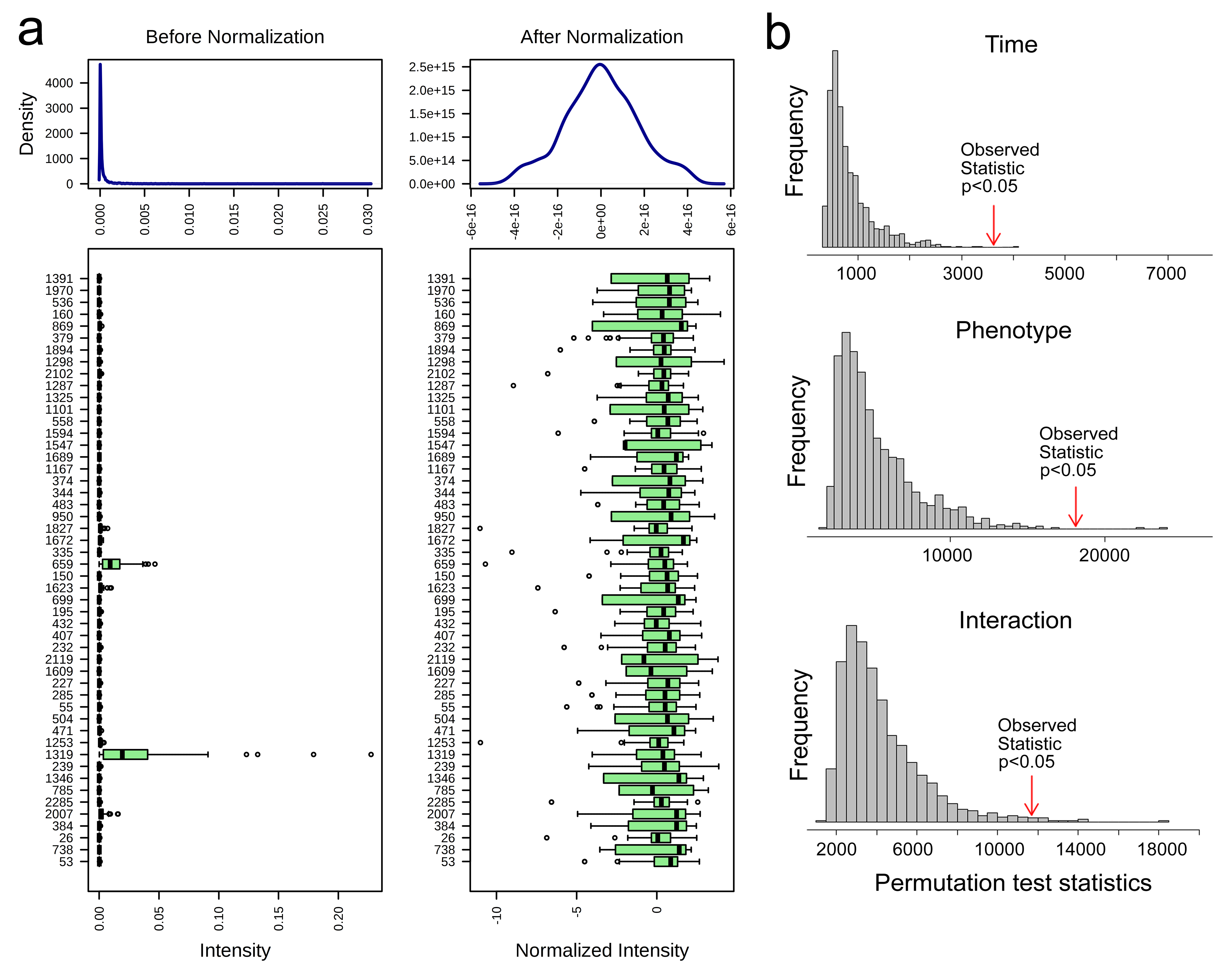

Supplement: Supplementary Figure 3 — Data normalization (A) and model validation (B) of the dynamic metabolomics data. The effect of data normalization was shown (A). Model validations were performed through permutations, as demonstrated by significance levels of p < 0.05 for the phenotype, time, and the interaction between phenotype and time. [file Image_3.TIF]
