# Supplementary material for: Short Term Intrarectal Administration of Sodium Propionate Induces Antidepressant-Like Effects in Rats Exposed to Chronic Unpredictable Mild Stress
Source: Front Psychiatry. 2018 Sep 27;9:454. doi: 10.3389/fpsyt.2018.00454 (PMC6170646; doi:10.3389/fpsyt.2018.00454)
Supplement: Supplementary Table 1 — Metabolites identified from fecal metabolome of rats in this study. [file Table_1.DOC]

| Supplementary Table 1. Metabolites identified from fecal metabolome of rats in this study. | | | | |
| --- | --- | --- | --- | --- |
| **Metabolites** | **HMDB ID** | **RT(min)** | **Ions(m/z)** | **Match (NIST)*** |
| L-Lactic acid | HMDB0000190 | 7.01 | 73, 128, 176 | 942 |
| Acetic acid | HMDB0000042 | 7.30 | 73, 142, 174 | 967 |
| Sarcosine | HMDB0000271 | 7.70 | 73, 246 | 794 |
| L-Alpha-aminobutyric acid | HMDB0000452 | 8.81 | 73, 147, 218 | 867 |
| Phosphoric acid | HMDB0002142 | 8.93 | 73, 218 | 805 |
| L-Valine | HMDB0000883 | 9.56 | 73, 204, 218 | 873 |
| Valeric acid | HMDB0000892 | 9.84 | 73, 147, 292, 333 | 861 |
| Caproic acid | HMDB0000535 | 10.05 | 73, 156 | 796 |
| Benzoic acid | HMDB0001870 | 10.38 | 73, 147, 233 | 925 |
| Glycerol | HMDB0000131 | 10.54 | 73, 129, 247 | 826 |
| L-Leucine | HMDB0000687 | 10.62 | 73, 129, 147, 205 | 862 |
| L-Isoleucine | HMDB0000172 | 11.04 | 99, 241, 256 | 858 |
| Glycine | HMDB0000123 | 11.36 | 73, 147, 245 | 890 |
| Succinic acid | HMDB0000254 | 11.50 | 73, 147, 217, 305 | 828 |
| Propanoic acid | HMDB0000237 | 11.72 | 73, 232 | 940 |
| Pyrimidine | HMDB0003361 | 12.03 | 73, 147, 174, 248 | 882 |
| Maleic acid | HMDB0000176 | 12.28 | 73, 144 | 865 |
| L-Serine | HMDB0000187 | 12.40 | 73, 147, 189 | 793 |
| L-Threonine | HMDB0000167 | 12.96 | 73, 241 | 948 |
| Glutaric acid | HMDB0000661 | 13.53 | 73, 147, 333 | 871 |
| L-Alanine | HMDB0000161 | 14.06 | 73, 158, 218 | 910 |
| Malic Acid | HMDB0000744 | 15.43 | 55, 73, 75, 149, 259 | 887 |
| L-Aspartic acid | HMDB0000191 | 16.27 | 73, 147, 261 | 830 |
| L-Methionine | HMDB0000696 | 16.34 | 73, 318 | 848 |
| L-Proline | HMDB0000162 | 16.45 | 75, 262, 337 | 862 |
| D-Fructose | HMDB0000660 | 17.56 | 73, 147, 164 | 839 |
| Pimelic acid | HMDB0000857 | 17.93 | 73, 117, 313 | 845 |
| Phenylacetic acid | HMDB0000209 | 18.67 | 73, 117, 129, 339 | 783 |
| L-Glutamic acid | HMDB0000148 | 18.84 | 73, 75, 147, 247 | 796 |
| Phenylalanine | HMDB0000159 | 19.13 | 77, 105, 135, 179 | 841 |
| L-Arabinose | HMDB0029942 | 20.19 | 73, 147, 159 | 865 |
| L-Fucose | HMDB0000174 | 21.46 | 73, 156 | 791 |
| Ornithine | HMDB0000214 | 23.91 | 73, 103, 159 | 849 |
| L-Lysine | HMDB0000182 | 24.87 | 73, 116 | 935 |
| D-Allose | HMDB0001151 | 25.77 | 73, 130 | 857 |
| Piperidinecarboxylic acid | HMDB0000070 | 26.45 | 73, 147 | 899 |
| Glucaric acid | HMDB0000663 | 26.77 | 73, 174, 200 | 788 |
| L-Tyrosine | HMDB0000158 | 26.81 | 73, 262, 292 | 878 |
| Gluconic acid | HMDB0000625 | 27.15 | 73, 147, 204, 319, 361 | 826 |
| 2-Indolecarboxylic acid | HMDB0002285 | 27.48 | 73, 299 | 847 |
| Palmitic acid | HMDB0000220 | 28.73 | 73, 147, 205 | 850 |
| N-Acetyl-D-glucosamine | HMDB0000215 | 29.08 | 73, 117, 147 | 857 |
| Myo-Inositol | HMDB0000211 | 29.25 | 73, 117 | 814 |
| Linoleic acid | HMDB0000673 | 31.27 | 73, 147, 205, 319 | 923 |
| Lactose | HMDB0000186 | 38.06 | 73, 147, 217 | 899 |

*Match quality against the NIST reference library
