# Supplementary material for: Short Term Intrarectal Administration of Sodium Propionate Induces Antidepressant-Like Effects in Rats Exposed to Chronic Unpredictable Mild Stress
Source: Front Psychiatry. 2018 Sep 27;9:454. doi: 10.3389/fpsyt.2018.00454 (PMC6170646; doi:10.3389/fpsyt.2018.00454)
Supplement: Supplementary Table 2 — Typical metabolites identified by the ASCA dynamic metabolomics analysis. [file Table_2.DOC]

| Supplementary Table 2. Typical metabolites identified by the ASCA dynamic metabolomics analysis. | | | | | | | |
| --- | --- | --- | --- | --- | --- | --- | --- |
| Model | Metabolite | Leverage | SPE |  |  |  |  |
| Time | Lactic acid | 2.00E-03 | 4.79E+00 |  |  |  |  |
|  | Valeric acid | 2.11E-03 | 4.18E+00 |  |  |  |  |
|  | Glycerol | 1.55E-03 | 6.91E+00 |  |  |  |  |
|  | Isoleucine | 1.59E-03 | 3.71E+00 |  |  |  |  |
|  | Serine | 1.75E-03 | 2.08E+00 |  |  |  |  |
|  | Valine | 1.65E-03 | 4.06E+00 |  |  |  |  |
| Phenotype | Lactic acid | 2.30E-03 | 3.65E-30 |  |  |  |  |
|  | Acetic acid | 2.41E-03 | 1.23E-30 |  |  |  |  |
|  | Hexanoic acid | 1.96E-03 | 6.16E-31 |  |  |  |  |
|  | Phosphoric acid | 2.49E-03 | 6.16E-31 |  |  |  |  |
|  | Succinic acid | 2.15E-03 | 6.16E-31 |  |  |  |  |
|  | Valeric acid | 2.22E-03 | 1.23E-30 |  |  |  |  |
|  | Malic acid | 2.04E-03 | 6.16E-31 |  |  |  |  |
|  | Glycerol | 2.29E-03 | 6.16E-31 |  |  |  |  |
|  | Glycine | 1.93E-03 | 6.16E-31 |  |  |  |  |
|  | Valine | 1.79E-03 | 7.70E-31 |  |  |  |  |
|  | Sarcosine | 2.94E-03 | 6.16E-31 |  |  |  |  |
|  | Serine | 1.74E-03 | 1.54E-31 |  |  |  |  |
|  | Threonine | 1.74E-03 | 3.08E-31 |  |  |  |  |
|  | Alanine | 2.83E-03 | 6.16E-31 |  |  |  |  |
|  | Aspartic acid | 1.62E-03 | 1.54E-31 |  |  |  |  |
|  | Pyrimidine | 1.86E-03 | 1.23E-30 |  |  |  |  |
| Interaction | Lactic acid | 3.02E-03 | 9.26E-01 |  |  |  |  |
|  | Propanoic acid | 2.60E-03 | 1.77E+00 |  |  |  |  |
|  | Valeric acid | 3.13E-03 | 5.92E+00 |  |  |  |  |
|  | Malic acid | 3.04E-03 | 7.22E+00 |  |  |  |  |
|  | Glycerol | 3.35E-03 | 5.81E+00 |  |  |  |  |
|  | Leucine | 2.38E-03 | 3.79E-01 |  |  |  |  |
|  | Glycine | 2.48E-03 | 1.44E+00 |  |  |  |  |
|  | Pyrimidine | 2.83E-03 | 2.01E+00 |  |  |  |  |
|  | Serine | 2.60E-03 | 2.22E-01 |  |  |  |  |
|  | Threonine | 2.52E-03 | 1.23E+00 |  |  |  |  |
|  | Methionine | 6.91E-03 | 5.02E+00 |  |  |  |  |
|  | Valine | 2.48E-03 | 8.39E+00 |  |  |  |  |
|  | Sarcosine | 3.23E-03 | 1.87E+00 |  |  |  |  |
